# Optimal Frequency and Amplitude of Vertical Viewpoint Oscillation for Improving Vection Strength and Reducing Neural Constrains on Gait

**DOI:** 10.3390/e23050541

**Published:** 2021-04-28

**Authors:** Wei Wang, Kaiming Yang, Yu Zhu

**Affiliations:** Beijing Key Laboratory of Precision and Ultra-Precision Manufacturing Equipment and Control, Department of Mechanical Engineering, Tsinghua University, Beijing 100084, China; pc_ustb_wang@163.com (W.W.); zhuyu@tsinghua.edu.cn (Y.Z.)

**Keywords:** gait complexity, viewpoint oscillation, self-motion illusion, virtual walking, multiscale entropy

## Abstract

Inducing self-motion illusions referred as vection are critical for improving the sensation of walking in virtual environments (VE). Adding viewpoint oscillations to a constant forward velocity in VE is effective for improving vection strength under static conditions. However, the effects of oscillation frequency and amplitude on vection strength under treadmill walking conditions are still unclear. Besides, due to the visuomotor entrainment mechanism, these visual oscillations would affect gait patterns and be detrimental for achieving natural walking if not properly designed. This study was aimed at determining the optimal frequency and amplitude of vertical viewpoint oscillations for improving vection strength and reducing gait constraints. Seven subjects walked on a treadmill while watching a visual scene. The visual scene presented a constant forward velocity equal to the treadmill velocity with different vertical viewpoint oscillations added. Five oscillation patterns with different combinations of frequency and amplitude were tested. Subjects gave verbal ratings of vection strength. The mediolateral (M-L) center of pressure (CoP) complexity was calculated to indicate gait constraints. After the experiment, subjects were asked to give the best and the worst oscillation pattern based on their walking experience. The oscillation frequency and amplitude had strong positive correlations with vection strength. The M-L CoP complexity was reduced under oscillations with low frequency. The medium oscillation amplitude had greater M-L CoP complexity than the small and large amplitude. Besides, subjects preferred those oscillation patterns with large gait complexity. We suggested that the oscillation amplitude with largest M-L CoP complexity should first be chosen to reduce gait constraints. Then, increasing the oscillation frequency to improve vection strength until individual preference or the boundary of motion sickness. These findings provide important guidelines to promote the sensation of natural walking in VE.

## 1. Introduction

Walking in VE has been reported to improve task performance [1], promote rehabilitation training engagement [2] and the degree of entertainment [3]. Walking in VE can be achieved by walking-in-place, redirection and reposition methods. Among these methods, reposition is highly desirable for providing real walking sense since it adopts a locomotion interface to neutralize the user’s walking displacement. A treadmill is a common locomotion interface to realize unidirectional walking in VE.

Apart from a specific locomotion interface, the designation of VE also plays an important role for providing truly immersive virtual walking experience. In daily walking, human visual system can register any type of self-motion on the basis of optic flow presented to the user. Herein, the optic flow is defined as the temporal change in the pattern of light intensities in different directions at the moving point of observation [4]. Based on this, the sensation of walking in VE can be improved by the movement of virtual object to provide the user with compelling illusions of self-motion. These illusions of self-motion are referred as vection [5].

Vection can be improved by changing the fidelity, scene rendering and contrast of VE. However, these kinds of vection improvements need high resolution display devices to make the most of it. A more reliable method to improve the sensation of walking is to introduce dynamic changes of the user’s viewpoint. For example, Stephen et al. [6] found that adding horizontal or vertical viewpoint jittering to a constant forward velocity can produce shorter vection onset and longer vection durations than non-jittering optic flow. The jittering advantage would not be affected by the jitter frequency, amplitude and direction. Adding viewpoint oscillations can also improve vection in VE. Different from viewpoint jittering, the oscillation advantage was influenced by frequency and direction. Specifically, adding vertical viewpoint oscillations would induce greater vection strength than horizontal viewpoint oscillations [7]. High frequency vertical oscillations would provoke stronger vection than oscillations with low frequencies [8]. However, these oscillation advantages are only confirmed under user static conditions [6,7,8].

During treadmill walking, viewpoint oscillation can produce greater perceived motion in depth speed compared with viewpoint jittering [9], therefore would lead to stronger vection. Investigating how the oscillation frequency and amplitude affect the vection strength under treadmill walking condition would help to strengthen virtual reality (VR) immersion. Therefore, the first purpose of this study was to investigate the relationship between the vertical oscillation frequency, amplitude and the user ratings of vection strength during treadmill walking. Since R. H. Y. et al. [10] have pointed out that the oscillation speed would be the major factor for inducing vection, the relationship between vection strength and vertical oscillation speed during treadmill walking was also investigated.

The existence of visuomotor entrainment mechanism [11,12] is another concern for improving the sensation of walking in VE. Due to the mechanism, human gait characteristics would be influenced when different visual oscillations are applied to VEs. For example, Jason et al. [11] perturbed a virtual hallway with different mediolateral driving frequencies, and found that these visual perturbations had frequency dependent effects on metrics of walking balance. Patricia et al. [12] reported that step length and step width variability were significantly increased under visual oscillation conditions than no oscillation conditions. These influences of visuomotor entrainment mechanism on human gait would eventually cause undesirable walking experiences and compromise VR immersion if the oscillation pattern was unproperly designed. Therefore, the other purpose of this study was to determine the effects of different vertical oscillation frequency and amplitude on gait complexity. Specifically, the mediolateral center of pressure (M-L CoP) signal was used to quantify gait complexity. To the best of author’s knowledge, no previous study has addressed these problems before.

The rest of the paper is organized as follows. Section 2 introduces the experiment setup and procedures. Details of signal processing and statistics are also provided. Section 3 presents the results of this study. Discussion and conclusion are presented in Section 4.

## 2. Materials and Methods

### 2.1. Participants

Seven healthy adults (age: 33.57 ± 4.47 year, height: 172.14 ± 2.54 cm, weight: 71.14 ± 10.98 kg) voluntarily participated in the experiment. All subjects have normal or corrected to normal vision, and no history of lower-limb or neuromuscular injury. They have never experienced VR treadmill walking before. Each subject provided informed consent and the institutional review board of Tsinghua University approved the experiment.

### 2.2. Experiment Setup and Procedures

#### 2.2.1. Experiment Setup

**Figure 1:**
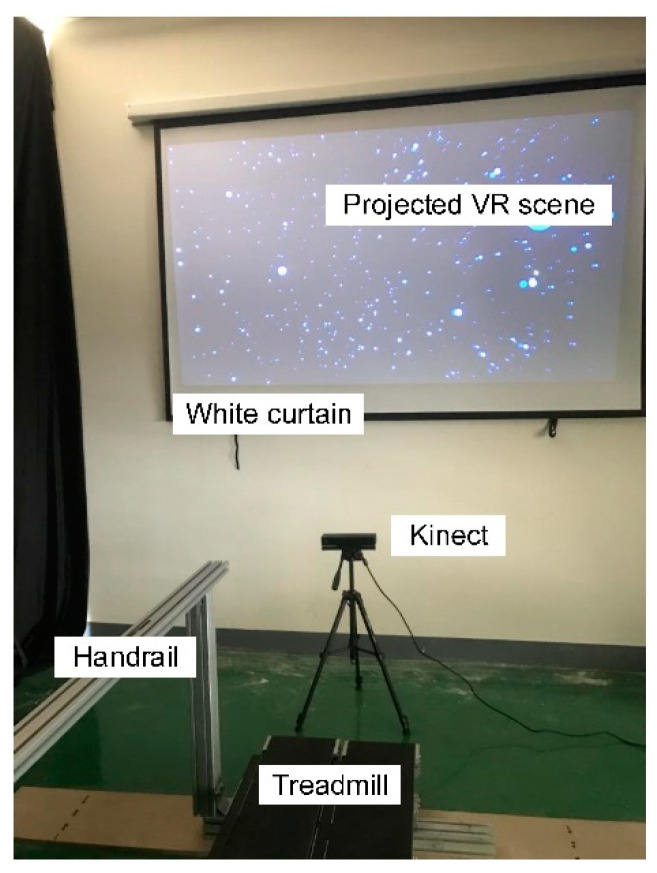
Experiment setup. Subjects can walk on the treadmill while viewing the VR scene. A handrail was placed on the left of the treadmill to ensure the safety during walking.

A custom-made treadmill system was used to allow subjects to walk on the treadmill at predetermined speeds. The treadmill system comprised of two belts and a handrail shown as in Figure 1. For each belt, a three-phase permanent magnet synchronous motor was incorporated to drive the motion of the belt. The speed command of the treadmill system was updated through an embedded ARM controller with a frequency of 100 Hz. The custom-made treadmill system has been proved valid in our previous works [13,14]. The Unity3D software was used to build the VR scene in Figure 1. The scene was consisted of blue balls with random locations. By adding sinusoid oscillation movements to these balls, they can simulate viewpoint oscillations and therefore enhance vection. Similar VEs have been used in several existing vection studies [15,16]. During treadmill walking, the VR scene was projected on the white curtain by a projector located at the rear of the treadmill.

To investigate the relationship between the oscillation frequency, amplitude and the strength of vection, five different vertical viewpoint oscillation patterns were specially designed as: y_1_ = 0.2 sin(2πt), y_2_ = 0.2 sin(2πt/1.5), y_3_ = 0.2 sin(2πt/0.5), y_4_ = 0.36 sin(2πt/0.5) and y_5_ = 0.04 sin(2πt/0.5). Noted that y_1_, y_2_, and y_3_ had the same oscillation amplitude but different frequencies, while y_3_, y_4_, and y_5_ had the same oscillation frequency but different amplitudes.

**Figure 2:**
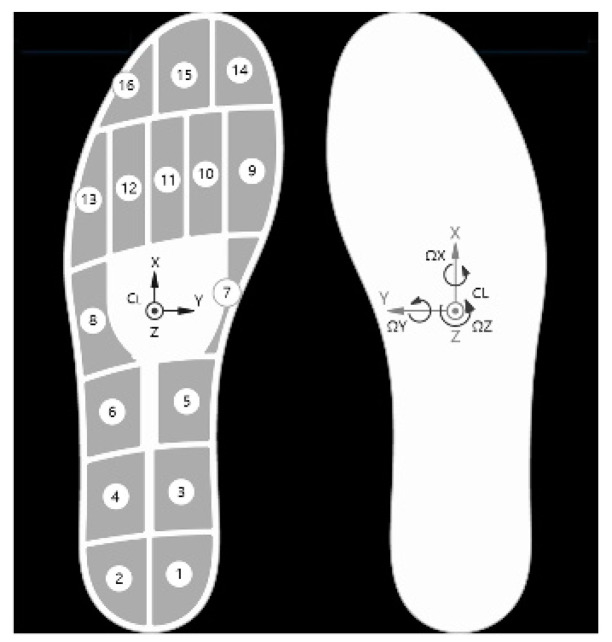
Sensor configuration of OpenGo insole pressure system. The left side presented the distribution of pressure sensors and the axis direction of IMU. The right side gave the definition of rotation direction.

An insole plantar pressure measuring device named OpenGo v2 was adopted to obtain the M-L CoP signal during treadmill walking. The OpenGo v2 system has 16 pressure sensors distributed across the insole embedded with a 6-axis inertial measurement unit located at the center. Details of pressure sensor locations can be found in Figure 2. The OpenGo v2 system can run at 10, 50, and 100 Hz which was chosen in this study. Additionally, the system has an internal flash memory to store real-time pressure data. An app version of Moticon software was installed in a Samsung mobile phone to connect with the OpenGo v2 system through Blue Tooth protocol. The pressure data can then be recorded by the mobile phone and wirelessly transferred to a computer installed with a desktop version of Moticon software. Since the OpenGo v2 system is cableless, it is highly desirable for measuring plantar pressure with no intrusiveness to user’s walking experience [17].

#### 2.2.2. Procedures

All Subject were given 3 min to habituate treadmill walking before the experiment. After that, the OpenGo v2 insoles were put into the subject’s shoes. The subject then stepped on the treadmill and stood still. The insole sensors of one foot were reset to zero when it was raised. Similar resetting procedure was done to the other foot.

During the experiment, the treadmill velocity was set as the subject’s preferred walking speed, and the optic flow speed in the depth direction equaled to the treadmill velocity. Subjects were asked to walk on the treadmill for 2 min while viewing the virtual scene with one of the five oscillation patterns added to the depth optic flow. They were required to look at the center of VE to avoid distraction during walking. The other four oscillation patterns were then tested in a random order. Subjects were given 1 min to rest before starting another trial.

Subjects gave verbal ratings of their perceived strength of vection in depth at the end of each 2 min walking. The rated range of vection strength was “0” to “10”. Defining the vection strength under no vertical oscillation condition as “5”, if the added oscillation pattern led to a greater vection strength than no oscillation condition, the vection strength should be rated larger than “5”; if not, the vection strength was rated smaller than “5”. During the vection rating process, the subject kept walking under no viewpoint oscillation condition until a vection rating was given.

After the vection rating procedure, subjects were required to give the best and the worst oscillation patterns based on their walking experience.

### 2.3. Signal Processing

The first and the last 10 s data were excluded from the M-L CoP to remove those signals induced by actions of start and stop walking. To improve the signal to noise ratio, the M-L CoP signal was reconstructed using empirical mode decomposition (EMD) algorithm [18,19]. Procedures of the EMD algorithm are depicted as follows.

Step 1: Detect the minima and maxima for a given signal *f*(*t*).

Step 2: Build two envelopes from these minima and maxima using cubic-spline interpolation.

Step 3: Calculate the mean value *m*(*t*) of the two envelopes.

Step 4: Subtract *m*(*t*) from the original signal *f*(*t*) to obtain a candidate as

(1) d(t)=f(t)−m(t)

Step 5: The above four steps are called sifting process and iterated until the mean value of *d*(*t*) is below a user-defined threshold. The selection of threshold has been fully addressed in [20]. Herein, a typical value of 0.05 was adopted.

Step 6: After k iterations, the threshold can be satisfied and the resulted signal is called an intrinsic mode function (IMF) depicted as

(2) $$c_{1}(t)=d_{k}(t)$$

Step 7: The corresponding residual can then be calculated as

(3) $$r_{1}(t)=f(t)-c_{1}(t)$$

Step 8: Iterating step 1 to 5 on the residual *r*_1_(*t*) to extract all the IMFs until the resulted residual is a constant, a monotonic slope or a function with only on extrema.

Based on the above, the original signal *f*(*t*) can be decomposed into several IMFs and a residual

(4) $$f(t)=\sum_{i=1}^{n}c_{i}+r_{n}$$

**Figure 3:**
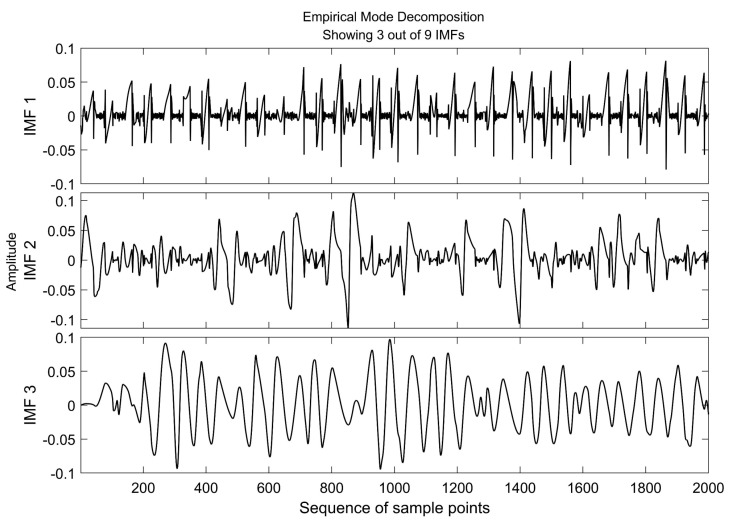
A typical EMD result of M-L CoP signal showing 3 out of 9 IMFs. The first (IMF 1) and the second (IMF 2) IMF were polluted by high frequency noise.

Noted that EMD does not require any selected input parameters or initial conditions. It is therefore an adaptive and robust method for dealing with gait signals of different individuals. In this study, the M-L CoP signal was reconstructed by eliminating the first and the second IMF from the original signal (Figure 3).

Multiscale entropy (MSE) was used to quantify the complexity of filtered M-L CoP signal. For a given time series {*x*_1_, *x*_2_, ……, *x*_N_}, the MSE method first constructed consecutive coarse-grained time series with different time scales as follows

(5) $$u_{j}^{\left(\tau \right)}=1/\tau \sum_{i=(j-1)\tau +1}^{j\tau }x_{i}$$

where *τ* represents the time scale factor and 1 ≤ *j* ≤ N/*τ*.

From each course-grained time series, sample entropy (SE) was calculated as the negative natural logarithm of the conditional probability *C*(*r*):

(6) $$SE(m,r,\tau )=-\ln\frac{C^{m+1}(r)}{C^{m}(r)}$$

where *m* denotes the length of a template vector and *r* represents the point matching tolerance. $C^{m+1}(r)$ is the number of template vector pairs having $d\left[u_{m+1}(i),u_{m+1}(j)\right]<r$, and $C^{m}(r)$ is the number of template vector pairs having $d\left[u_{m}(i),u_{m}(j)\right]<r$. Herein, d[  ] denotes the Chebyshev distance of given vector pairs.

**Figure 4:**
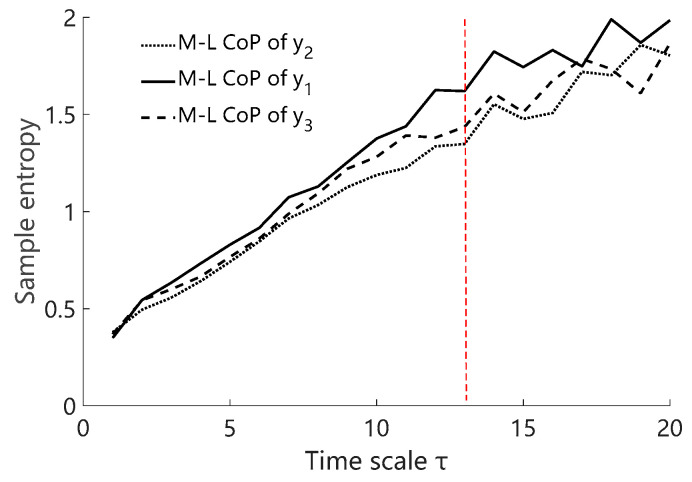
An example of SE changing with time scale *τ* for filtered M-L CoP data of y_1_, y_2_, and y_3_ oscillation pattern. The SE values on the right side of the red dotted line exhibited abrupt changes, which meant the SE values were no longer reliable.

Parameter selection of MSE was conducted based on the rules in [21]. As a result, the template vector length *m* equaled to 2; the point matching tolerance *r* equaled to 0.2 times the signal standard deviation; the largest analyzed time scale *τ* was determined as 13 where the SE of subsequent time scales became unstable (Figure 4).

Finally, a complexity index (*CI*) was calculated as the area under the curve of SE versus. *τ* to provide an indication of overall complexity:

(7) $$CI=\sum_{i=1}^{\tau }SE(i)$$

where a higher *CI* value indicates greater complexity.

### 2.4. Statistics

Pearson correlations were used to assess the relationship between the oscillation frequency, amplitude, speed and the rated vection strength. The oscillation speed was calculated as the oscillation frequency times the amplitude. Consequently, five different oscillation speeds derived from y1 to y5 were calculated as S1 = 0.2, S2 = 0.13, S3 = 0.4, S4 = 0.72, S5 = 0.08, respectively. A correlation coefficient of 0.5 to 0.7 indicates a moderate correlation, and greater than 0.7 proves a strong correlation. Besides, vection strength was linearly fitted with the oscillation frequency, amplitude and speed based on least squares estimate to determine which factor best predicted the vection strength. The principle of least squares estimate was to minimize the sum of squared error between the actual and the estimated value. Take the rated vection strength and oscillation frequency for example, the linear regression equation was obtained as

(8) $$\begin{array}{l} vs_{est}=a_{0}\cdot f+b_{0}\\ a_{0}=\sum (f_{i}-\bar{f})(vs_{i}-vs_{avg})/\sum {\left(f_{i}-\bar{f}\right)}^{2}\\ b_{0}=vs_{avg}-a_{0}\bar{f} \end{array}$$

where $vs_{avg}$ denotes the mean actual vection strength, and $\bar{f}$ denotes the mean oscillation frequency. $vs_{i}$ and $f_{i}$ represents the actual vection strength and frequency of *i*-th sample, respectively. $vs_{est}$ is the estimated vection strength.

In this study, the dependent variable was the CI value of M-L CoP signal. Factors that may have effects on the dependent variable were the oscillation frequency, amplitude and speed. Two-way ANOVA with one replication per cell was performed for each of the three factors after testing the dependent variable for normal distribution. Confident intervals of 95% were also given for each condition, with effect size estimated using Cohen’s *f* statistic (<0.1, small effect; around 0.25, medium effect; >0.4, large effect). For factors that have significant effects on the CI of M-L CoP, post hoc pairwise comparisons using least significant difference method were performed.

All statistical processing was completed using SPSS Statistics (IBM Corp., Somers, NY, USA), and the significance level was set as 0.05.

## 3. Results

**Figure 5:**
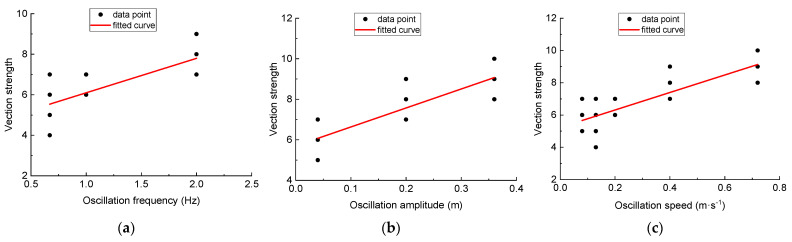
The linear correlations of vection strength with (**a**) oscillation frequency: vection rating = 1.7 × frequency + 4.4, (**b**) oscillation amplitude: vection rating = 9.4 × amplitude + 5.7, (**c**) oscillation speed: vection rating = 5.4 × speed + 5.2.

Pearson correlation analysis and linear curve fitting results proved that there was a strong positive correlation between the oscillation frequency and the vection strength (*r* = 0.764, *p* < 0.001) shown in Figure 5a. The vection strength also displayed a strong positive correlation with the oscillation amplitude (*r* = 0.875, *p* < 0.001) and speed (*r* = 0.840, *p* < 0.001) shown in Figure 5b,c, respectively.

The oscillation frequency, amplitude and speed had significant effects on the complexity of M-L CoP (*F* = 10.509, *p* = 0.002 for frequency; *F* = 28.012, *p* < 0.001 for amplitude; *F* = 8.245, *p* < 0.001.), all with a large effect size (See Table 1).

**Figure 6:**
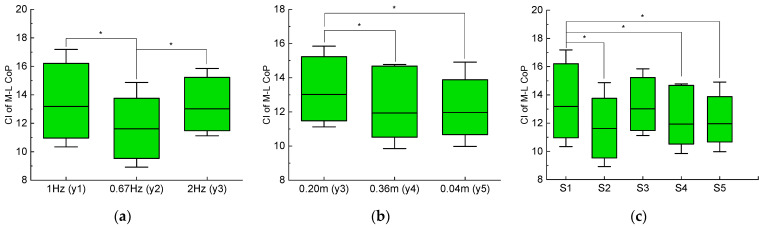
Post hoc comparisons of (**a**) oscillation frequency, (**b**) oscillation amplitude, and (**c**) oscillation speed effects on the complexity of M-L CoP. Significant differences between two groups are connected using solid lines with an asterisk (*) above.

The post hoc results showed that for different oscillation frequencies, the M-L CoP complexity of y2 oscillation pattern was significantly smaller than that of y1 (*p* = 0.001) and y3 (*p* = 0.003) pattern. The complexity between y1 and y3 pattern was not different. As for oscillation amplitudes, the M-L CoP complexity of y3 pattern was significantly larger than that of y4 (*p* < 0.001) and y5 (*p* < 0.001) pattern. There was no difference between the complexity of y4 and y5 pattern. For oscillation speeds, the M-L CoP complexity of S1 speed was significantly larger than that of S2 (*p* < 0.001), S4 (*p* = 0.002) and S5 (*p* = 0.002) speed. Besides, the M-L CoP complexity of S3 speed was significantly larger than that of S2 (*p* < 0.001), S4 (*p* = 0.005) and S5 (*p* = 0.006) speed, and smaller than that of S1 speed but not significant (mean difference = −0.161, *p* = 0.648). The complexity of S2, S4 and S5 speed were not different from each other. Details can be found in Figure 6.

Table 2 summarized the best and the worst oscillation patterns given by subjects. Four subjects rated y1 pattern as the best, and two subjects rated y3 as the best. y5 was rated as the best pattern by one of the seven subjects. Additionally, six subjects rated y4 as the worst pattern plus one who rated y2 as the worst pattern.

## 4. Discussion

This study aimed at determining the optimal oscillation frequency and amplitude for improving vection strength and reducing gait constraints. We first investigated the relationships between user rated vection strength and the vertical viewpoint oscillation characteristics during treadmill walking. The results showed that the oscillation frequency, amplitude and speed all had strong positive correlations with vection strength. Thus, increasing oscillation frequency and amplitude would induce stronger vection strength during treadmill walking. These findings were partly consistent with the work of Stephen et al. [8] and with that of R.H.Y., et al. [10]. According to Figure 4, the estimated vection strength under no oscillation condition was “4.4” for frequency, “5.7” for amplitude and “5.2” for oscillation speed. Since the predefined vection strength under no oscillation condition was “5”, it can be inferred that the vection strength under no oscillation condition can be more accurately estimated from the relationship between vection strength and oscillation speed than from that of oscillation frequency and amplitude. Taken together, we suggested that the oscillation speed would be the major factor to affect vection strength. Noted that our results were obtained under treadmill walking condition, while the work of Stephen et al. [8] and R. H. Y. et al. [10] focused on vection under static conditions.

Another important finding of this study was that the viewpoint oscillation characteristics had significant effects on the complexity of M-L CoP. According to the finding in [22,23], the M-L CoP complexity was an indicator of constraints exerted by central nervous system on locomotor behavior, with greater complexity indicating less constraints. Based on our findings, changing viewpoint oscillation characteristics would then induce different degrees of constraints on locomotor behavior. Herein, the M-L CoP complexity was reduced under the lowest oscillation frequency (0.67 Hz) compared with other high frequencies (1 Hz and 2 Hz). This indicated that a low oscillation frequency would introduce more constraints on gait than a high frequency. Since low oscillation frequencies would also reduce vection strength, we suggested the choice of high oscillation frequencies for enabling natural walking in VE. For the oscillation amplitude, the largest M-L CoP complexity existed at the medium amplitude value (0.2 m). Too large (0.36 m) or too small (0.04 m) oscillation amplitude would introduce more constraints on gait. As a result, despite larger oscillation amplitude would induce greater vection strength, it would come at the cost of more gait constraints.

In this study, y1 oscillation pattern was preferred by four subjects, followed by y3 preferred by two subjects. Besides, y1 and y3 pattern had significantly greater complexity than other patterns. These findings were supported by the work of Gregory et al. [24], who suggested that walking at a preferred condition usually exhibits higher complexity than a non-preferred condition. Similarly, Damasio et al. [25]. found that walking at preferred speed caused larger stride time complexity than non-preferred walking speeds. Consequently, the subject’s preference can be used as a guider when searching for the best viewpoint oscillation amplitude and frequency to reduce neural constraints on gait.

As a result, to achieve stronger vection with less constraints on gait, the oscillation amplitude with the largest M-L CoP complexity should be chosen. After determining the oscillation amplitude, it was suggested to increase the oscillation frequency for improving vection strength until reaching the user’s preference or the edge of motion sickness symptoms. Our findings provide valuable basis to induce the perception of real walking in VE. Future work should focus on applying the optimal viewpoint oscillation to a feedback-controlled treadmill.

## Figures and Tables

**Table 1:** ANOVA results. * denoted a significant effect. A large effect size was bolded.

Dependent Variable	Oscillation Pattern	Mean (std)	95% Confidence Interval		*F*	*p*	*η* ^2^
			Lower Bound	Upper Bound			
CI of M-L CoP	frequency	**12.60** (**2.25**)	**11.58**	**13.63**	10.509	0.002 *	**0.637**
	amplitude	12.31 (1.87)	11.45	13.16	28.012	<0.001 *	**0.824**
	speed	12.34 (2.10)	11.62	13.06	8.245	<0.001 *	**0.579**

**Table 2:** Subjects reported results about the best and the worst oscillation patterns.

Oscillation Pattern	y1	y2	y3	y4	y5
Best	4	0	2	0	1
Worst	0	1	0	6	0

## Data Availability

The data presented in this study are available on request from the corresponding author. The data are not publicly available due to privacy.

## References

[1] Roy A.R., Simon L. (2009). The benefits of using a walking interface to navigate virtual environments. ACM Trans. Comput. Hum. Interact..

[2] Tyler R., Chang S.N., Nedel L. (2018). Immersion of virtual reality for rehabilitation—Review. Appl. Ergon..

[3] Menin A., Torchelsen R., Nedel L. (2018). An analysis of VR technology used in immersive simulations with a serious game perspective. IEEE Comput. Graph. Appl..

[4] Gibson J.J. (1966). The Senses Considered as Perceptual Systems.

[5] Gibson J.J. (1950). Perception of the Visual World.

[6] Stephen P., Barbara J.G., Shane G.B. (2000). Global-perspective jitter improves vection in central vision. Perception.

[7] Anatole L., Jean M.B., Jean M.H., Donikian S. (2006). Camera motions improve the sensation of walking in virtual environments. Proceedings of the IEEE Virtual Reality Conference.

[8] Stephen P., Frederick B., Andrea B., John F. (2007). Vertical display oscillation effects on forward vection and simulator sickness. Aviat. Space Environ. Med..

[9] April A., Stephen P., Deborah A., Robert S.A. (2013). Vection in depth during treadmill walking. Perception.

[10] So R.H.Y., Wei Y., Chen D.J.Z. (2017). Vection provoked by visual oscillation: Is frequency the major determining factor. Proceedings of the IEEE 6th Global Conference on Consumer Electronics.

[11] Jason R.F., Carrie A.F., Matthew S.A., Darryl G.T. (2017). Visuomotor entrainment and the frequency-dependent response of walking balance to perturbations. IEEE Trans. Neural Syst. Rehabil. Eng..

[12] Patricia M.M., Jonathan B.D., Jason M.W. (2010). Walking variability during continuous pseudo-random oscillations of the support surface and visual field. J. Biomech..

[13] Wang W., Yang K., Zhu Y., Mu H. (2020). Speed adaptation and acceleration ripple suppression of treadmill user system using a virtual force moment balance model. Trans. Inst. Meas. Control.

[14] Wang W., Yang K., Zhu Y., Qian Y., Wan C. (2020). A comparison of variability and gait dynamics in spatiotemporal variables between different self-paced treadmill control modes. J. Biomech..

[15] Deborah A., Stephen P. (2014). The role of perceived speed in vection: Does perceived speed modulate the jitter and oscillation advantages?. PLoS ONE.

[16] Stephen P., Bernhard E.R. (2018). The search for instantaneous vection: An oscillating visual prime reduces vection onset latency. PLoS ONE.

[17] Wei W., Kai M.Y., Zhu Y., Qian Y., Wan C., Li M. (2021). Walking speed estimation from a wearable insole pressure system embedded with an accelerometer using Bayesian neural network. ASME J. Med. Diagn..

[18] Norden E.H., Zheng S., Steven R.L., Manli C.W., Hsing H.S., Quanan Z., Nai C.Y., Chi C.T., Henry H.L. (1998). The empirical mode decomposition and the Hilbert spectrum for nonlinear and non-stationary time series analysis. Proc. R. Soc. Lond. A.

[19] Zhao H.W., Norden E.H. (2009). Ensemble empirical mode decomposition: A noise-assisted data analysis method. Advan. Adapt. Data Analy..

[20] Flandrin P., Goncalves P., Rilling G. (2004). Detrending and denoising with empirical mode decomposition. Proceedings of the 12th European Signal Processing Conference.

[21] Brian J.G., Chung K.P., Peter M.W., Andrew C.A. (2015). Multiscale entropy analysis of center of pressure dynamics in human postural control: Methodological considerations. Entropy.

[22] James T.C., Vicki S.M., Nicholas S. (2009). Approximate entropy detects the effect of a secondary cognitive task on postural control in healthy young adults: A methodological report. J. NeuroEng. Rehabil..

[23] Ben Y.L., Fu L.W., Chi W.L., Xue Y.Z., Xiao L.W., Yih K.J. (2019). Complexity-Based Measures of Postural Sway during Walking at Different Speeds and Durations Using Multiscale Entropy. Entropy.

[24] Gregory S.W., Zoe T. (2019). Complexity, symmetry and variability of forward and backward walking at different speeds and transfer effects on forward walking: Implications for neural control. J. Biomech..

[25] Damasio M.C., Peng K.C., Ary L.G., Jeffrey M.H. (2003). Multiscale entropy analysis of human gait dynamics. Physica A Statis. Mecha. Appl..

